# Distribution and habitat use patterns of the endangered Central American clouded oncilla (*Leopardus pardinoides oncilla*) in Costa Rica

**DOI:** 10.1371/journal.pone.0310562

**Published:** 2024-09-17

**Authors:** José D. Ramírez-Fernández, Lester A. Fox-Rosales, Michael S. Mooring, Juan Carlos Delgado-Carazo, Steven R. Blankenship, Jennifer R. Powell, Yoryineth Méndez, Angie Acevedo-Loría, Esteban Brenes-Mora, James G. Sanderson, Tadeu G. de Oliveira

**Affiliations:** 1 Oncilla Conservation, Costa Rica Wildlife Foundation, Montes de Oca, San José, Costa Rica; 2 Tiger Cats Conservation Initiative, São Luís, Brazil; 3 Departamento de Biologia, Universidade Estadual do Maranhão, São Luís, Maranhão, Brazil; 4 Point Loma Nazarene University, San Diego, California, United States of America; 5 Quetzal Education & Research Center, San Gerardo de Dota, San José, Costa Rica; 6 Laboratorio de Genética de la Conservación, Escuela de Biología, Universidad de Costa Rica, San José, Costa Rica; 7 Centro de Investigación en Biología Celular y Molecular, Universidad de Costa Rica, San José, Costa Rica; 8 Department of Physical and Environmental Sciences, University of Toronto Scarborough, Toronto, Ontario, Canada; 9 Research Program, Tropical Science Center, Monteverde, Puntarenas, Costa Rica; 10 Re:Wild, Austin, Texas, United States of America; 11 Small Wild Cat Conservation Foundation, Corrales, Nuevo México, United States of America; 12 Instituto Pro-Carnívoros, Atibaia, São Paulo, Brazil; Kerala University of Fisheries and Ocean Studies, INDIA

## Abstract

Montane cloud forests are highly threatened ecosystems that are vulnerable to climate change. These complex habitats harbor many species that suffer the negative consequences of this global phenomenon, such as shifts in their distribution and habitat use. The Central American clouded oncilla (*Leopardus pardinoides oncilla*) is the smallest and most endangered wild cat in Mesoamerica and is primarily reported in cloud forests throughout its distribution. The species is poorly understood, with no studies conducted in Central America assessing its habitat preferences. To bridge this knowledge gap, we sampled two mountain ranges in Costa Rica with camera traps and conducted an occupancy analysis to understand the anthropogenic and environmental features that influence oncilla habitat use within them. Additionally, we conducted spatial predictions of habitat use across its northern and southern range in Costa Rica to identify priority conservation areas for the species. We found that Central American clouded oncilla habitat use is driven primarily by environmental factors. Our results showed that oncillas select habitats with denser tree cover at high elevations, closer to permanent water sources, which may provide them with high prey density and a favorable habitat structure for their survival. Spatial predictions identified two main regions as conservation priority areas where threat mitigation efforts and monitoring should be implemented: the Caribbean slope of the Talamanca mountains, and the Arenal-Monteverde forest complex. The occupancy modeling approach turned out to be very useful to assess the spatial associations of the species with the environment and mapping the conservation priority areas. Future research and mitigation actions should focus on potential threats that could negatively impact Central American clouded oncilla populations and habitat use, including the role of mesopredators and feral species.

## Introduction

**Fig 1:**
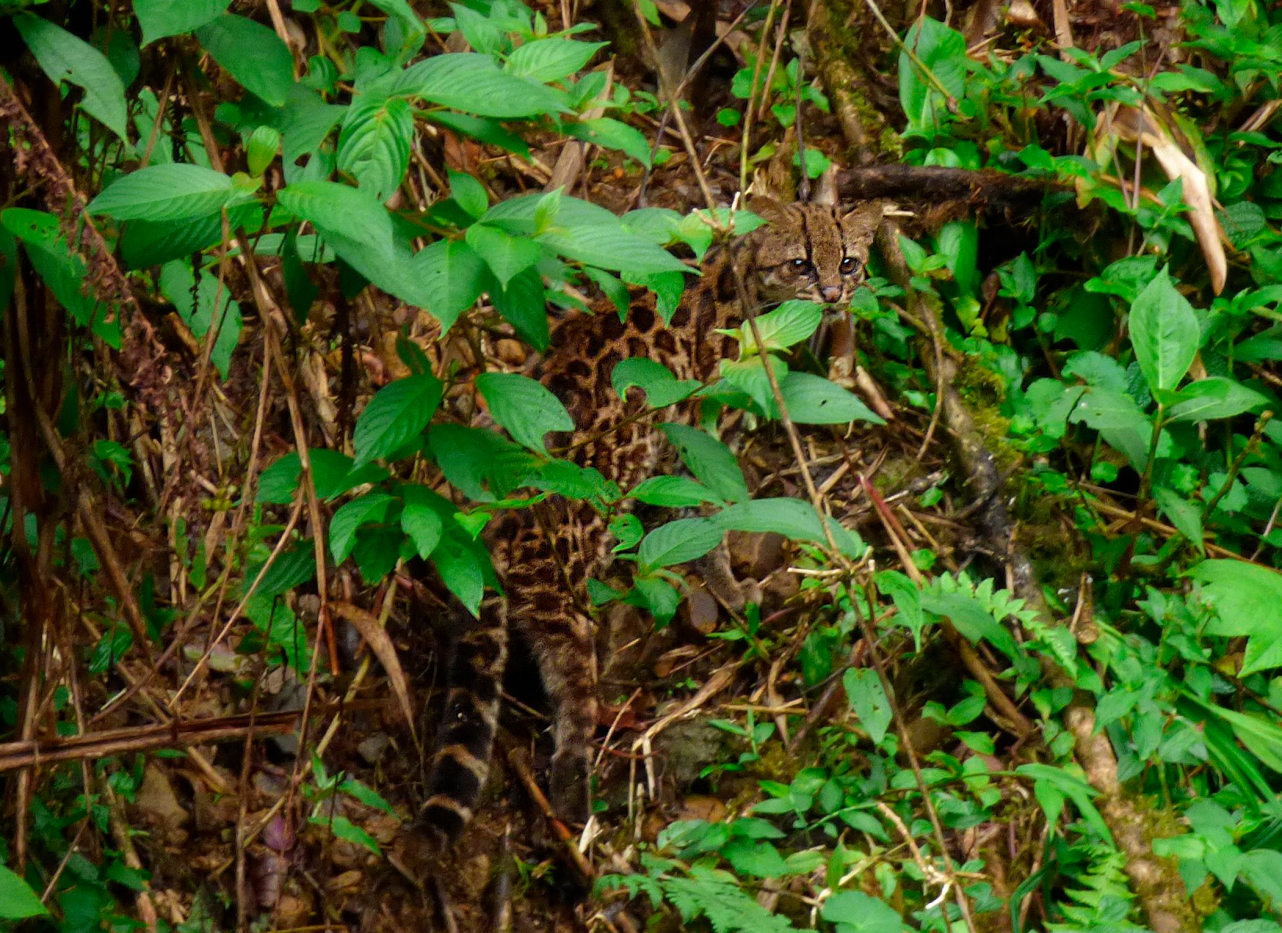
The Central American clouded oncilla (*Leopardus pardinoides oncilla*) inhabits the cloud forests and highlands of Costa Rica and western Panama.

**Fig 2:**
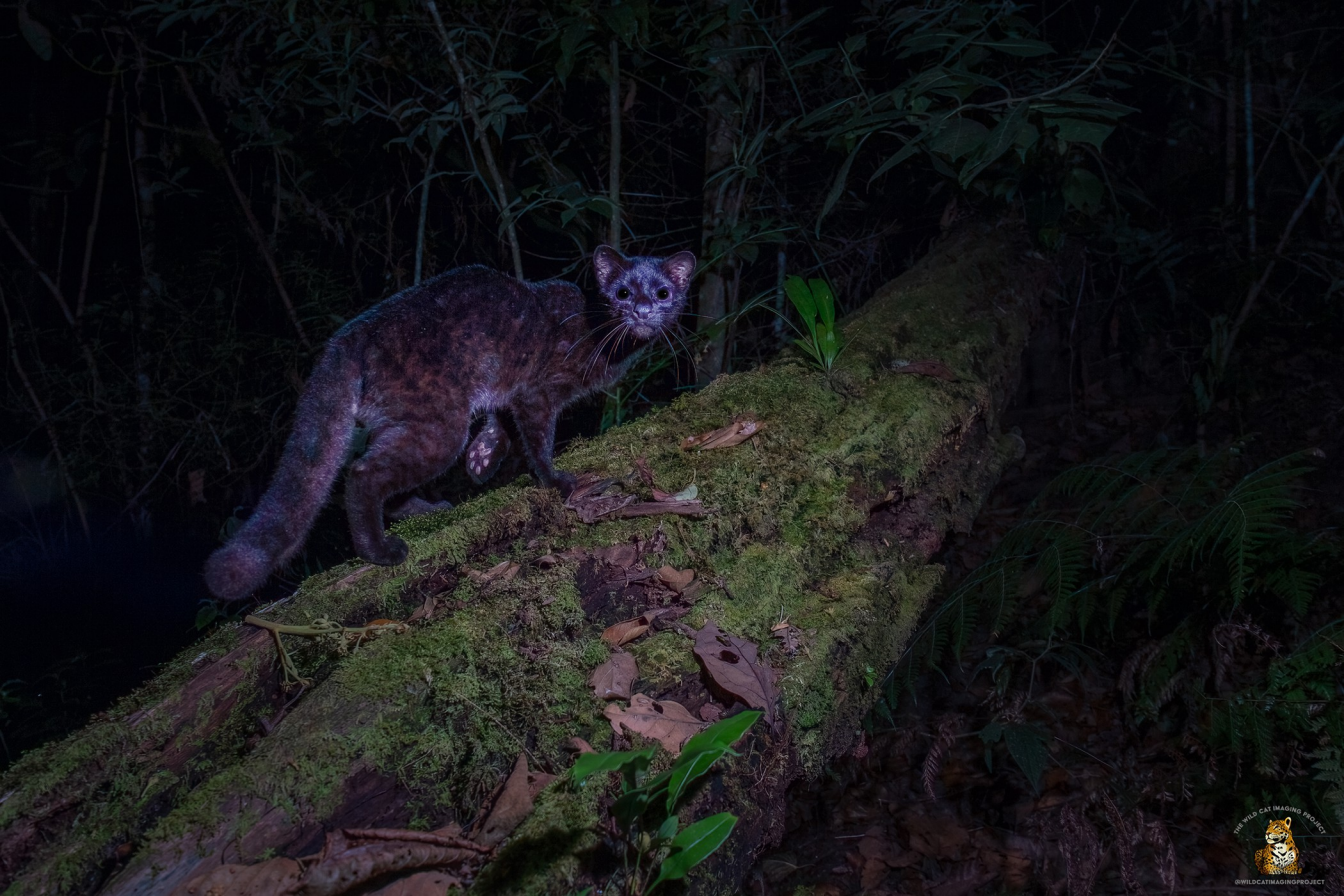
A melanistic individual of the Central American clouded oncilla in the Talamanca mountain range, Costa Rica.

The Central American clouded tiger-cat or oncilla (*Leopardus pardinoides oncilla*, Figs 1 and 2) is part of the *Leopardus* “tigrina” species complex. It was recently suggested that the Central American clouded oncilla is a subspecies of the clouded tiger-cat (*L*. *pardinoides*), a former subspecies of the complex which was recently elevated to species level [1]. The clouded tiger-cat ranges discontinuously from the southern Central American montane forests of Costa Rica and Panama, through the Andean chain of South America, to northwestern Argentina. The Central American subspecies is the smallest (1.5–3.5 kg), rarest, and most endangered wild cat in all Mesoamerica [1–4]. Categorized as “Vulnerable” in the IUCN Red List under *L*. *tigrinus* [5], and as “Endangered with extinction” by Costa Rican environmental authorities [6, 7]. Across its distribution it has been associated with different habitats, although Central American populations have been reported to occur mainly in montane cloud forest [2, 5].

These tropical montane ecosystems are known to be highly threatened by climatic change [8–10]. Increases in temperature, dry season length, and number of dry days are just a few examples of the consequences produced by climate change in tropical highlands [11]. In Costa Rica, the negative effects of this global phenomenon have been modeled for highland amphibians, reptiles, and birds [11–13]. This situation forces many middle elevation species to change their distribution to higher elevations, thereby changing species composition and interactions in the altitudinal gradient [14, 15].

Cloud forests are particularly vulnerable to climate change because (1) their existence requires specific environmental conditions [16–18], (2) they have a natural patchy distribution [19, 20], and (3) much of their original distribution has already disappeared [21, 22]. It has been suggested that the ecological complexity of cloud forests makes them an “archipelago in the highlands” [23]. Considering that these ecosystems are under strong pressure from human activities, have great hydrological value [24, 25], high biodiversity and endemism [20, 23], montane cloud forests must be prioritized for conservation.

The Central American clouded oncilla is not only the most endangered wild cat in Mesoamerica, but also an endemic subspecies restricted to a highly susceptible and fragile habitat in Costa Rica and Panama. Because felid predators play a key role in regulating prey populations and structuring animal communities, establishing their current distribution and abundance is a priority issue for conservation action. The application of modelling techniques for estimating species distributions is a crucial tool for assessing the environmental factors driving the occurrence of rare species such as the oncilla. There are several approaches to model species distributions, with some techniques using presence only data and others using presence absence data. These models have been used to delineate species distributions at a global scale [1] and make predictions of future distribution under climate change scenarios [26]. A major issue in modeling the distribution of elusive species is accounting for detectability during the sampling stage. Failure to account for lack of detection when a species is present, may result in less precise inferences of species distribution and habitat associations [27]. Occupancy models estimate the distribution of a species while accounting for imperfect detection, by modeling the detectability of the species at a given site and thereby reducing the risk of false absences [28]. This makes occupancy analysis useful for assessing the drivers of species occurrence while distinguishing between ecological and observational processes.

Regarding the “tigrina” species complex split after de Oliveira et al. [1], two clouded tiger-cat subspecies are recognized, the Andean clouded tiger-cat *L*. *pardinoides pardinoides* in South America and the clouded oncilla *L*. *pardinoides oncilla* in Central America. Research on the clouded tiger-cat (under *L*. *tigrinus*) has been performed on South American populations in order to understand its ecological requirements and monitor its populations [29–32], allowing to identify areas where conservation efforts should be enforced for this species to thrive. However, for the Central American populations no research has been implement on these subjects.

In this study, we identified the environmental features that had the greatest influence on habitat use by Central American oncilla in Costa Rica; we also predicted oncilla habitat use in two regions of the country, enabling conservation efforts to be allocated appropriately. We tested the following hypotheses, oncilla habitat use is: (1) mainly driven by vegetation structure and topography; (2) negatively influenced by anthropogenic activity; and (3) driven by a combination of environmental and anthropogenic features.

## Materials and methods

### Study site

**Fig 3:**
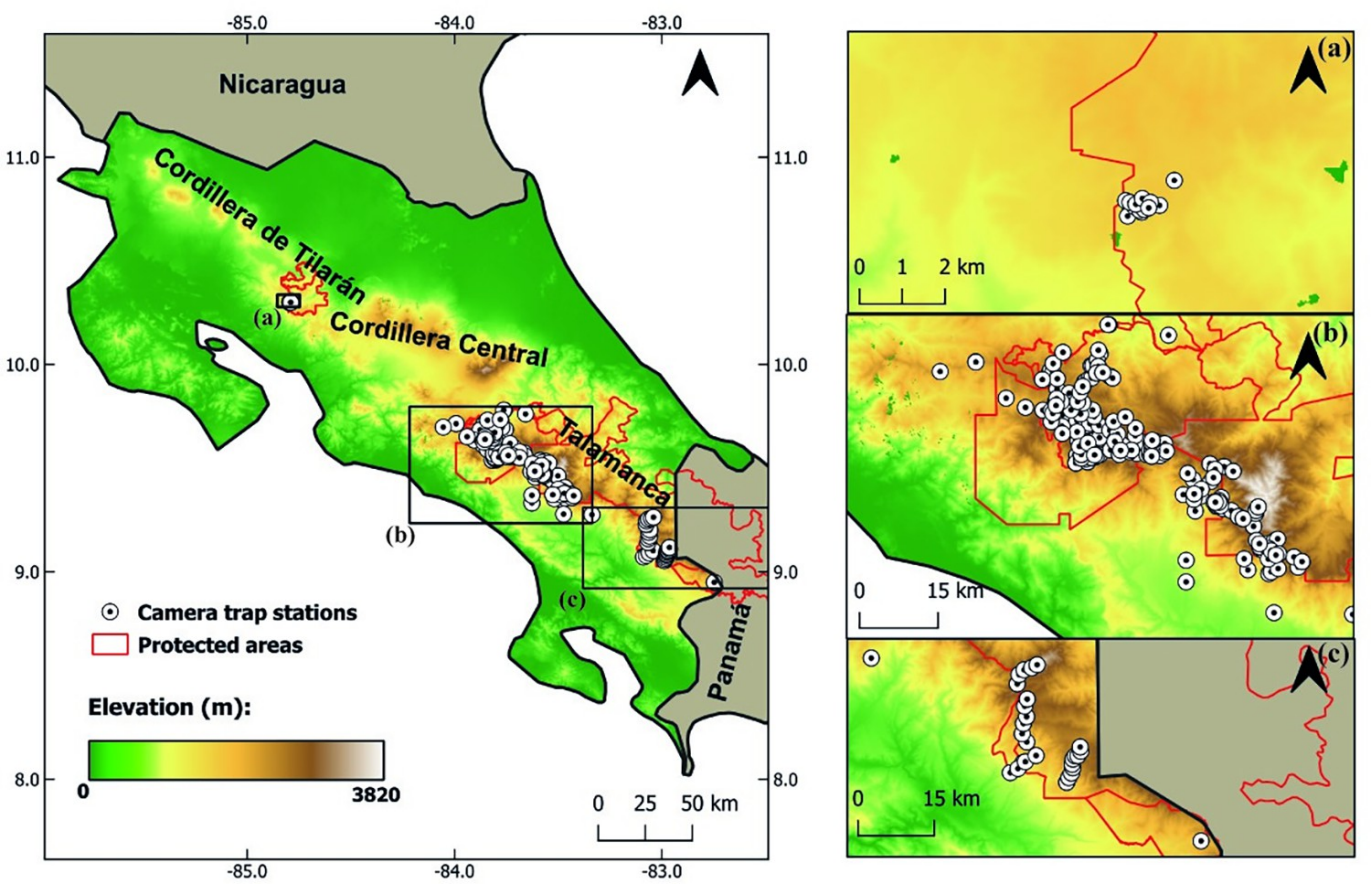
Study area and camera trap station locations in Costa Rica mountains. Base imagery derived from the United States Geological Survey, Digital Elevation–Shuttle Radar Topography Mission (SRTM) (https://www.usgs.gov/).

The study was conducted in two mountain ranges in Costa Rica spanning protected and unprotected areas (Fig 3). In the Arenal-Monteverde Protected Zone, within the Tilarán mountain range, in the northwest part of the country, we surveyed the Monteverde Cloud Forest Preserve. In central and southern Costa Rica, we sampled several protected areas of the Talamanca mountain range: Chirripó National Park (CNP), Los Quetzales National Park, Tapantí Macizo de la Muerte National Park (TMMNP), La Amistad International Park, and Las Tablas Protected Zone, and a mix of private reserves and unprotected areas within the CNP and TMMNP buffer zones. Vegetation sampled at most sites consisted of montane cloud forest, with some areas of elfin forest and paramo habitat.

Climate at all sites consisted of a dry (December-May) and wet season (June-November), and differences in rainfall between sites, with the Talamanca region being wetter than Arenal-Monteverde (S1 Table).

Because camera traps are a non-invasive tool with no animal captures involved, this study did not required clearance from an animal ethics board. This study is framed under MINAE and CONAGEBIO research permits (resolutions SINAC-ACC-PI-R-044-2020, R-SINAC-PNI-ACLAP-028-2020, and R-001-2021-OT-CONAGEBIO).

### Occurrence records

We compiled all the published data and available unpublished records of oncillas in Costa Rica between 2011 and 2022, including camera trap data, live records with photographic evidence, and carcasses. The records of oncilla were compiled with location details and GPS coordinates of each sighting. For this study, we used our own data (Oncilla Conservation–OC) and gathered additional camera trap data from four collaborators: (1) Tropical Science Center Monteverde (MV); (2) Nai Conservation (NAI); (3) Cloudbridge Nature Reserve (CNR); and (4) Quetzal Education & Research Center (QERC). We compiled a total of 712 independent records of clouded oncilla. Records were checked for accurate species identification to avoid common mistaken identities with margays (*Leopardus wiedii*) and ocelots (*Leopardus pardalis*).

### Sampling design

We set 65 camera trap stations in specific areas of the Talamanca mountain range where we did not have data from collaborators and where habitat characteristics matched reports for oncilla in Costa Rica. The exact location of the camera trap stations was based on accessibility. Excluding stolen and damaged cameras, we collected data from 53 camera trap stations from 2019 to 2022 for the analysis. Prior to installation of cameras, we removed vegetation in the camera detection field that could interfere with the motion sensor to reduce the number of "ghost photos" caused by windblown vegetation. At each station, a single camera trap was placed at a height of ca. 40 cm on a natural (game) trail created by recent wildlife activity (e.g., tapir trails). We used several brands and models of passive infrared camera traps for each sampling station (BUSHNELL ® Trophy Cam HD Essential and Trophy Cam HD Aggressor, and BROWNING ® Strike Force HD Max and Dark Ops HD Max). We set the cameras to take a sequence of three photos with the minimum delay possible (0–5 s).

A similar sampling design from MV, NAI, CNR, and QERC databases is detailed in [33–36], respectively. Because camera traps are a non-invasive tool with no animal captures involved, this study did not require clearance from an animal ethics board.

### Covariates

We assessed a total of eight covariates to model oncilla habitat preferences (Table 1). This included six anthropogenic and environmental covariates to model the occupancy parameter: a) percent tree cover; b) distance to permanent water bodies; c) distance to nearest paved road; d) elevation; e) terrain ruggedness index; f) distance to nearest settlement (Table 1). Based on prior studies of oncilla and other tropical felids [32, 37–39], we hypothesized that oncillas would prefer areas with denser vegetation cover, higher elevation, more rugged terrain, and areas further away from roads and settlements. To model the detection parameter, we measured two additional covariates: g) camera trapping effort; and h) survey (Table 1). We hypothesized that a greater trapping effort would result in a higher detection probability. The “Survey” covariate was included to account for unmodeled heterogeneity between survey methods and study sites in the different databases (S1 Dataset).

**Table 1:** Covariates used for modelling oncilla detection probability and occupancy.

Covariates	Description	Mean (±SD)
**Detection (p)**		
Effort	Number of days cameras were active per occasion	13.15 (±4.23) days
Survey	Survey (categorical). To account for heterogeneity among databases and/or study sites	MV, NAI, CNR, QERC, Oncilla Conservation (OC)
**Occupancy (*Ψ*)**		
Tree cover	Percentage of vegetation taller than 5 m in a 50-m buffer around each camera	86.13 (±14.26) %
Distance to permanent water bodies	Euclidean distance to nearest permanent water body.	485.4 (±365.73) m
Distance to nearest paved road	Euclidean distance to nearest paved road	2513.36 (±2837.4) m
Elevation	Mean elevation above sea level in a 50-m buffer around each camera	2489.4 (±516.97) m
Terrain ruggedness	Measure of elevation variability in a 50-m buffer around each camera	25.43 (±12.08) m
Distance to settlements	Euclidean distance to nearest hamlet, village, town, or city	4875.55 (±4314.09) m

All covariates were z-transformed prior to model construction to allow direct comparisons and facilitate model convergence. We conducted Spearman correlations to examine collinearity between covariate pairs and considered covariates to be correlated whenever ρ ≥ |0.70|. All spatial covariate data were extracted using QGIS [40]. Elevation and terrain ruggedness were obtained from a Shuttle Radar Topography Mission digital elevation model [41]; tree cover data were derived from the Global Forest Change dataset [42]; distance to settlements came from the OpenStreetMap database and includes cities, towns, villages, and hamlets [43]; distance to paved roads and permanent water bodies were derived from the Costa Rican National Geography Institute [44].

### Occupancy modeling

We modeled oncilla occupancy using single-season, single-species occupancy models [28]. These models estimate two parameters: the probability that the species is present at a sampled site (occupancy, ψ), and the probability that the species is detected at a site given that it is present (detection, p). Because oncillas are a highly mobile species and our sampling was done with camera traps, we interpret oncilla occupancy as the probability of habitat use [45]. We first built a detection history matrix, using an occasion length of 15 days, with the package ‘camtrapR’ [46] in the R programming language ver. 4.2.3 [47].

We used up to 120 days from each camera trap station to meet the population closure assumption [48]. Because our surveys were carried out over several years and some of the sites were sampled more than once, we adopted a stacked approach whereby each unique camera-year combination was treated as a sampling site [49].

Occupancy models were implemented in a Bayesian modelling framework using the R package ‘ubms’ [50]. We ran several models, each representing a biologically relevant hypothesis. Initially we ran a null model, representing the hypothesis that oncilla occupancy and detectability do not vary along any of the 8 covariates. We also ran a null model with Year in the occupancy parameter to explore possible changes in occupancy with year. Then we ran two versions of the most parameterized model, including all 6 environmental predictors of the occupancy parameter and both the Effort and Survey measures of the detection parameter. One of these models included a random effect of camera trap station on the occupancy parameter to account for possible pseudoreplication from our stacked approach. The other model did not include this random intercept. Lastly, we ran a model using only environmental variables (testing hypothesis 1) and another model with only anthropogenic variables (testing hypothesis 2). To obtain the most parsimonious model, we ran versions deleting one parameter at a time (the parameter with the smallest effect size) to select the best model. We ran models using four Markov chains of 40,000 iterations each, with half discarded for burn-in. We used default non-informative priors. We ranked models using expected log pointwise predictive density (elpd). We calculated elpd using leave-one-out cross-validation for pairwise model comparisons. We assessed model fit by running the MacKenzie and Bailey goodness of fit test on the global model [51]. Additionally, we assessed model convergence by observing Rhat statistics > 1.05 and by visually examining the trace plots.

### Spatial predictions

Using the results of our occupancy modeling, we predicted oncilla occupancy across our study sites and the protected areas within them. We divided the area into 500x500 m grids to match the scale of the modelling and extracted covariate information for each grid cell. We then projected our most parsimonious occupancy model to predict oncilla habitat use probability in each grid cell.

## Results

After a total sampling effort of 59,687 trap-nights, we obtained a total of 253 independent detection events of oncilla.

**Fig 4:**
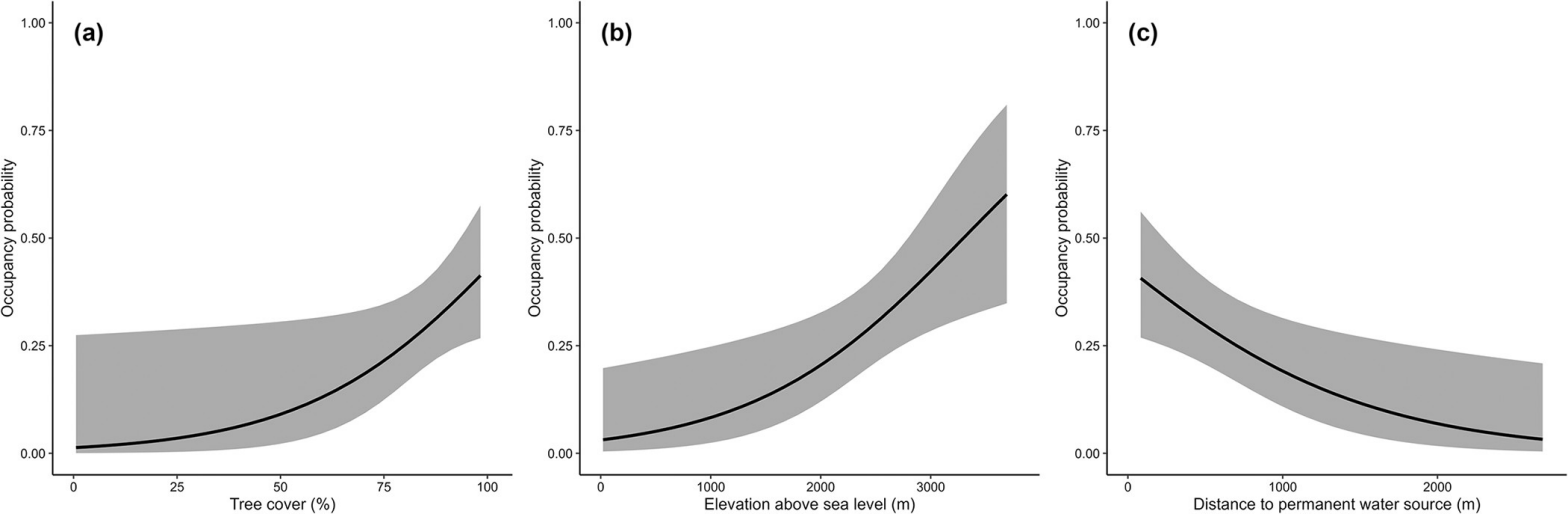
Central American clouded oncilla response to environmental covariates in the cloud forests of Costa Rica. Habitat use probability in response to (a) tree cover percentage; (b) elevation above sea level; and (c) distance to permanent water sources.

**Fig 5:**
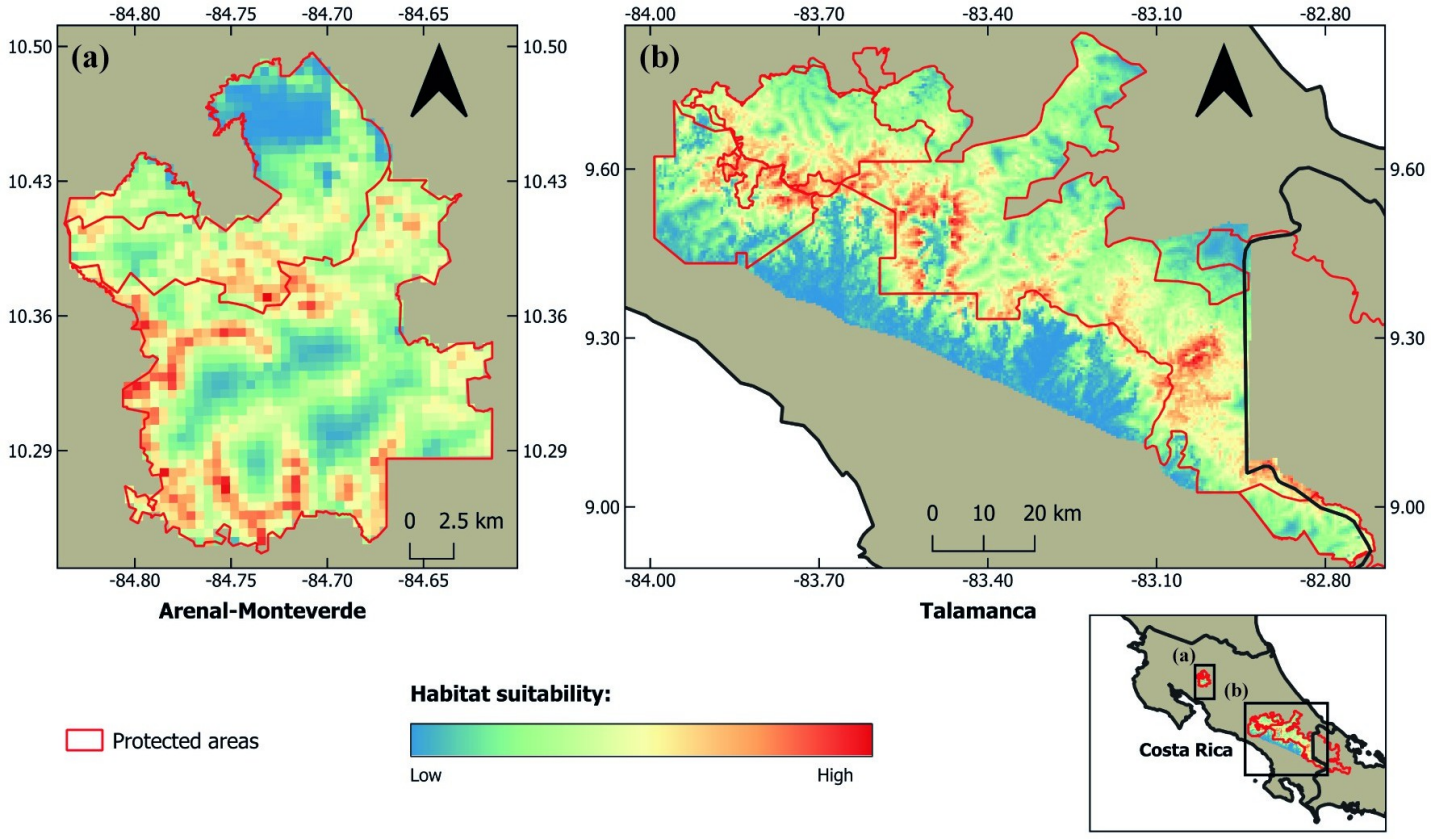
Habitat suitability for the Central American clouded oncilla in (a) the Arenal-Monteverde forest complex and (b) the Talamanca mountain range in northern and southern Costa Rica.

The models with the highest predictive accuracy included the random intercept effect for Station and did not include Year, suggesting static habitat use for the species during the sampling period (Table 2). The best model suggests that oncilla habitat use is most strongly influenced by tree cover (β = 0.69 ± 0.32, 95% CI = 0.06–1.32), elevation (β = 0.68 ± 0.26, 95% CI = 0.17–1.20), and distance to nearest water source (β = -0.42 ± 0.18, 95% CI = -0.76 –-0.07) (Fig 4), and to a lesser extent by terrain ruggedness (β = 0. 23 ± 0.20, 95% CI = -0.18–0.63). Although the coefficient suggests a positive effect on oncilla occupancy, terrain ruggedness had the smallest effect size and its 95% confidence interval included zero. Detectability was higher with increased survey effort (β = 0.34 ± 0.14, 95% CI = 0.06–0.62). Derived parameter estimates were 0.30 ± 0.05 (95% CI: 0.21–0.41) for habitat use and 0.09 ± 0.02 (95% CI: 0.06–0.13) for detection. Our global model performed well under the Mackenzie and Bailey goodness of fit test (Chi square = 1130.91, *P* = 0.3). Spatial predictions suggest high habitat suitability for oncilla in the Talamanca mountains and to a lesser extent the Tilarán mountain range, with the highest suitability in Tilarán in the core zone of the Children’s Eternal Rainforest, which we did not survey (Fig 5). In the Talamanca range, suitability was high throughout the Caribbean slope and lower in the Pacific slope (Fig 5).

**Table 2:** Model selection table. Models are ranked by expected predictive accuracy (elpd); P denotes the detection parameter and ψ the habitat-use parameter.

Model	elpd	Δelpd	Model weight
P(effort)ψ(tc+water+elev+tri)	-490.19	0.00	0.96
P(effort)ψ(set+road)	-495.39	-5.21	0.04
P(effort)ψ(global)^a^	-495.96	-5.77	0.00

^a^Global model includes all covariates.

## Discussion

We have conducted the first extensive analysis of the Central American clouded oncilla habitat use, thus shedding light on its ecological preferences at the northern portion of its range. Our analysis revealed that environmental features influence oncilla habitat use, supporting hypothesis 1. Our results indicate that oncillas prefer areas of dense tree cover at higher elevations, close to permanent water sources; there was little to no influence of terrain ruggedness, distance to roads, and distance to settlements on habitat use.

Tree cover emerged as the main environmental driver of oncilla occupancy, with the species occupancy increasing in denser areas. This pattern has also been seen for tiger-cat populations in the Brazilian drylands [37, 52]. Within the mountainous landscapes of the Talamanca range, vegetation cover varies from lowland rainforest to cloud forest at middle elevations, and elfin forest and paramo at high elevations. The latter habitat would be unsuitable for oncilla according to our results. However, we did record oncillas in the paramo at areas surrounded by oak forest or nearby oak trees which provide enough cover but were not tall enough to be categorized as a forest in the satellite imagery used in our analysis [42]. A preference for denser vegetation cover has also been documented for Andean clouded tiger-cats and Atlantic Forest tiger-cats [1]. Even the savanna tiger-cat shows a preference for denser vegetation within the savannas and semi-arid dry shrub-woodlands [38, 52, 53].

Small mammals, especially rodents, are reported as the *Leopardus* “tigrina” species complex’s main prey [5, 54–56], and denser areas are likely to provide both cover and a prey base. Particularly in the Talamanca mountain forests, mice are significantly more abundant in areas of denser vegetation and greater environmental complexity (i.e., oak forest) than in paramo [57]. Thus, the habitat use pattern of oncilla appears to be concentrated in areas of higher rodent abundance. In terms of prey diversity, the Talamancan deermouse (*Peromyscus nudipes*) (an endemic species included in the oncilla diet [54]) is the most abundant small mammal in this region [57]. Felid populations elsewhere have been shown to increase their habitat use in areas of higher prey density [58, 59]. However, we note the need to establish long-term standardized surveys of rodent abundance and diversity along with camera trap monitoring of small wild cats to evaluate the effect of rodent abundance on the habitat use of oncilla.

We found a linear relationship in which the probability of oncilla habitat use increased with elevation. In the tropical Andes, clouded tiger-cat populations have been shown to exhibit greater occupancy at elevations around 2500 meters above sea level [32]. The mountain ranges in which Central American clouded oncillas occur do not reach the high elevations of the Andes (> 4000 m), with the highest summits in Talamanca averaging 3600–3800 m above sea level, which is within the maximum known limit recorded for clouded tiger-cats (3960 m) [1], and those in the Tilarán all below 1900 m above sea level. The bulk of natural habitat in both mountain ranges is represented by upland cloud forest, which is the oncilla’s most important habitat. Hence, the positive effect of elevation on the species’ habitat use may be related to favorable habitat structure at mid and high elevations. At higher elevations there is also less human disturbance, which is especially true in the Talamanca mountain range. We note that some oncilla detections occurred at elevations of more than 3200 m, not far from Costa Rica’s highest summit (3820 m).

Distance to nearest water source was an important determinant of oncilla habitat use, with occupancy increasing in areas near water sources. This pattern contrasts with that found in savanna tiger-cats [37, 53]. The effect size of distance to water sources was smaller than both tree cover and elevation, suggesting that this predictor is the least important of the three. Due to high rainfall, small creeks and other water sources not visible in satellite imagery are likely to be present in several areas of the oncilla’s range. Additionally, the cloud forest environment is very humid. This suggests that water availability per se is not a limiting factor for the species. The perceived effect of this variable might be an artifact of the sampling design, given that forested remnants in non-protected areas are mainly concentrated around riparian vegetation, which is protected by Costa Rican law. As a result, camera trap stations in non-protected areas were mainly located around these riparian zones. Nevertheless, riparian areas could enhance resource availability for small mammals like rodents, which in turn would provide a prey base for oncillas.

We found no evidence of terrain ruggedness influencing oncilla habitat use, although the coefficient suggests a positive effect. Since oncillas are cloud forest specialists, we anticipated a positive relationship between habitat use and both elevation and topographic ruggedness. Although topographic ruggedness has not been tested on savanna tiger-cat populations, it has been shown to positively affect occupancy of other felids elsewhere [60, 61]. Further sampling in less rugged terrain may show evidence of terrain ruggedness positively influencing oncilla occupancy. Alternatively, it could be possible that the species favors high elevation environments with lower ruggedness. Rugged terrain presents challenges for locomotion and is energetically more expensive compared with movement over trails and natural walkways. Felid species generally prefer moving along trails, and oncillas specifically have been suggested to use trails made by tapirs [34]. Such trails tend to be located in less rugged areas, which may explain the apparent lack of response of oncillas to terrain ruggedness.

Oncilla habitat use did not change in relation to distance to roads, with the species neither avoiding nor selecting areas near roads. This suggests that the species may be at risk of roadkill mortality on roads that cross the forests. For instance, Central America’s main road, the Pan-American highway, passes close to some of our sampling sites and serves as a border between two of Costa Rica´s major highland protected areas: Tapantí Macizo de la Muerte, and Los Quetzales National Parks. Oncillas are known to be killed on this and nearby roads [62]. Roads have been documented to affect the habitat use of felids elsewhere (e.g., [32, 62–65]). Alternatively, in the Caatinga shrubland and thorn forest, savanna tiger-cat occupancy did not vary in response to distance to the nearest road [37]. Roads facilitate access of poachers to remote areas, and even in otherwise pristine forests, roads often lead to widespread habitat loss and poaching. Although deforestation was not a major threat at our study sites, roads still pose a serious problem for oncillas due to the risk of collision mortality. In addition, poachers who are otherwise hunting for meat could use roads to kill oncillas opportunistically. Because the bulk of the oncilla range in the Talamanca mountains is not crossed by roads, roadkill mortality is perhaps not a range-wide threat. Still, as many as three oncillas per year are being killed along the Pan-American highway in Costa Rica [62].

Although we had expected a negative effect of human settlements on oncilla habitat use, this variable was poorly supported in the models. This result is in contrast to studies conducted in the Brazilian Caatinga [37] and Colombian Andes [32]. Some characteristics of our sampling procedures could potentially explain this. All of our cameras were located in fairly pristine areas, and the vast majority were inside protected areas. Furthermore, a large portion of our cameras were in difficult-to-access terrain. Roughly 93% of our camera trap stations were more than 1 km away from the nearest settlement, with some as far away as 20 km. For tiger-cat populations in the Brazilian drylands, 800-m from the nearest household has been documented as a critical distance affecting the species’ habitat use [52]. If this pattern also holds true for oncilla, we expect a marked negative effect on habitat use in areas less than 800-m away from the nearest settlement. Alternatively, oncillas are known to occasionally prey on poultry by attacking chicken coops in local communities [66], thus oncillas in human-dominated landscapes might select habitats closer to settlements if these provide a foraging resource. Retaliatory killing is known to be a threat for some oncilla populations, as happens with other small wild cat species [67]; hence, we would expect a negative effect of distance to settlements on the species’ abundance more than on habitat use per se. For some areas, management schemes and the tolerance of local residents could explain a lack of response to settlements. At a private reserve in the Brazilian Caatinga, for example, tiger-cat habitat use did not change with distance to households, likely because the tiger-cats are not persecuted there [53].

Our spatial predictions suggest that the stronghold for the species in Costa Rica lies in the Talamanca mountains, with high suitability throughout Chirripó, Los Quetzales, and Tapantí National Parks and La Amistad International Park. High suitability was also found in the Arenal-Monteverde forest complex, but this area is smaller (48,273 ha.). Our maps suggest high suitability on the Caribbean slope of the Talamanca mountains, a relatively unexplored area that warrants further field surveys. On the Pacific slope of the mountains, suitability is lower as a result of past deforestation. Suitability was also found to be low in the highest area of Chirripó National Park, coinciding with the open paramo. In this area, oncillas could still be present, finding cover and prey in shrubby vegetation that does not qualify as proper tree cover. In-situ vegetation metrics such as litter depth and basal area could further elucidate oncilla habitat use in the paramo. In the Andes, litter depth was found to strongly influence clouded tiger-cat occupancy [32].

The use of occupancy modeling allowed us to identify areas of suitable habitat for the species, and hence delineate conservation priority areas for the species in the country. By leveraging a large camera trap dataset, we were able to account for unequal sampling effort and imperfect detection in an occupancy modeling framework. Lack of accounting for sampling effort and detectability can lead to biased estimates of habitat suitability using other modeling approaches [68]. A limitation of our approach is that we did not sample the full range of covariates, as cameras were often placed in areas where we expected a priori to find clouded oncillas. Nevertheless, the large number of sampling units and the geographic spread of sample sites allowed us to account for most of the species’ proposed range in the country. Our spatial prediction of habitat suitability matches that of de Oliveira et al. [1], which identified the Talamanca mountain range as the species’ stronghold in Central America.

## Conclusion and future directions

We have shown that Central American clouded oncilla habitat use patterns in the northern portion of its range are driven primarily by environmental factors. Future research should address the influence of mesopredators, such as the ocelot, on the habitat use patterns of the oncilla. Ocelots have been documented to negatively affect the populations of small Neotropical felids [69]. However, as the ocelot is mostly a lowland species, its numbers in clouded tiger-cat ranges are typically too small to cause an effect, as has been noted in Colombia [32, 70]. We also do not know if ocelot numbers could be limiting clouded tiger-cats to higher altitudes as it seems to do for savanna tiger-cats in Amazonia [69]. Additionally, assessing and mitigating other threats such as the presence of feral dogs and domestic cats is imperative due to the potential threat of disease transmission [52, 67]. Although most of the current suitable habitat for the unique Central American clouded oncilla falls under some form of protection, threats such as long-term climate change and disease transmission could wreak havoc on oncilla populations in the future. Density estimates are also much needed, as so far none have been published for *L*. *pardinoides*. Lastly, field surveys on the Panamanian side of the Talamanca mountains and in Costa Rica’s central mountain range are needed to fully delineate the scope of the conservation needs/requirements, status, and strategic conservation planning for this unique felid population of Central America.

## Supporting information

S1 Table Sampled areas to assess occupancy and habitat use patterns by the Central American clouded oncilla (*Leopardus pardinoides oncilla)* in the mountains of Costa Rica. (DOCX)

S2 Table Covariate correlation matrix. There were no correlations, hence all covariates were used in the modeling.
(DOCX)

S1 Dataset Site covariates data used for running the analysis of this manuscript. (XLSX)

S2 Dataset Observation covariates data used for running the analysis of this manuscript. (XLSX)

S3 Dataset Detection history matrix used for running the analysis of this manuscript. (XLSX)
