# Supplementary material for: Distribution and habitat use patterns of the endangered Central American clouded oncilla (Leopardus pardinoides oncilla) in Costa Rica
Source: PLoS One. 2024 Sep 17;19(9):e0310562. doi: 10.1371/journal.pone.0310562 (PMC11407673; doi:10.1371/journal.pone.0310562)
Supplement: S1 Table — (DOCX) [file pone.0310562.s001.docx]

**Table S1. Sampled areas to assess occupancy and habitat use patterns by the Central American clouded oncilla (*Leopardus pardinoides oncilla)* in the mountains of Costa Rica.**

| Site | Camera trap stations | Elevational range (m) | Annual temperature range (°C) | Annual precipitation range (mm) | Size (ha.) |
| --- | --- | --- | --- | --- | --- |
| Chirripó National Park (CNP) | 81 | 1400 – 3821 | 6.0 – 15.0 | 2548 – 4852 | 50,340 |
| Tapantí-Macizo de la Muerte National Park (TMMNP) | 118 | 1220 – 2560 | 7.6 – 18.2 | 2346 – 5881 | 58,495 |
| CNP Buffer Zone | 54 | 680 – 3135 | 6.0 – 23.3 | 2549 – 5241 | 34,772 |
| TMMNP Buffer Zone | 4 | 960 – 2500 | 13.5 – 19.8 | 2998 – 3751 | 2,052^a^ |
| La Amistad International Park | 89 | 2068 – 3317 | 7.5 – 19.8 | 2710 – 5223 | 199,147 |
| Las Tablas Protected Zone | 2 | 1876 – 1988 | 16.2 – 16.5 | 2455 – 2463 | 19,062 |
| Río Macho Forest Reserve | 29 | 2313 – 2756 | 7.8 – 11.8 | 2379 – 2652 | 22,572 |
| Los Santos Forest Reserve | 289 | 2108 – 3347 | 6.7 – 15.8 | 2367 – 4168 | 62,000 |
| Monteverde Cloud Forest Preserve | 28 | 860 – 1840 | 17.8 – 18.5 | 2843 – 2997 | 4,125 |
| Ujarrás Indigenous Reserve | 3 | 1730 – 2123 | 14.9 – 16.9 | 2121 – 2556 | 20,300 |
| Los Quetzales National Park | 23 | 1240 – 3190 | 8.4 – 12.6 | 2412 – 2968 | 4,117 |
| Cerro Vueltas Biological Reserve | 30 | 2600 – 3156 | 11.2 – 12.0 | 2349 – 2466 | 793.27 |

^a^This area corresponds only to three private lands sampled within the TMMNP Buffer Zone: La Marta Wildlife Refuge, Finca Queverí, and El Copal Finca Ecológica.
