# Supplementary material for: Distribution and habitat use patterns of the endangered Central American clouded oncilla (Leopardus pardinoides oncilla) in Costa Rica
Source: PLoS One. 2024 Sep 17;19(9):e0310562. doi: 10.1371/journal.pone.0310562 (PMC11407673; doi:10.1371/journal.pone.0310562)
Supplement: S2 Table — There were no correlations, hence all covariates were used in the modeling. (DOCX) [file pone.0310562.s002.docx]

**Table S2. Covariate correlation matrix.** There were no correlations, hence all covariates were used in the modeling.

|  | **WATER** | **ROAD** | **TC** | **ELEV** | **TRI** | **SET** |
| --- | --- | --- | --- | --- | --- | --- |
| **WATER** | - | 0.03 | 0.04 | 0.36 | -0.19 | -0.18 |
| **ROAD** |  | - | -0.20 | 0.36 | -0.08 | 0.60 |
| **TC** |  |  | - | -0.09 | -0.09 | -0.13 |
| **ELEV** |  |  |  | - | -0.34 | -0.12 |
| **TRI** |  |  |  |  | - | 0.25 |
| **SET** |  |  |  |  |  | **-** |
